# Is vancomycin MIC creep a worldwide phenomenon? Assessment of *S*. *aureus* vancomycin MIC in a tertiary university hospital

**DOI:** 10.1186/1756-0500-6-65

**Published:** 2013-02-19

**Authors:** Silvestre Joana, Póvoa Pedro, Gonçalves Elsa, Martins Filomena

**Affiliations:** 1 Polyvalent Intensive Care Unit, São Francisco Xavier Hospital, CHLO, Lisbon, Portugal; 2 CEDOC, Faculty of Medical Sciences, New University of Lisbon, Lisbon, Portugal; 3 Microbiology Laboratory, Clinical Pathology Department, São Francisco Xavier Hospital, CHLO, Lisbon, Portugal

## Abstract

**Background:**

Vancomycin is the primary treatment for infections caused by methicilin-resistant Staphylococcus aureus (MRSA). The association of vancomycin treatment failures with increased vancomycin minimum inhibitory concentration (MIC) is a well-recognized problem. A number of single-centre studies have identified progressive increases in glycopeptide MICs for *S. aureus* strains over recent years – a phenomenon known as vancomycin MIC creep. It is unknown if this is a worldwide phenomenon or if it is localized to specific centers.

**Methods:**

The aim of this study was to evaluate the trend of vancomycin MIC for isolates of MRSA over a 3-year period in a tertiary university hospital in Portugal. MRSA isolates from samples of patients admitted from January 2007 to December 2009 were assessed. Etest method was used to determine the respective vancomycin MIC. Only one isolate per patient was included in the final analysis.

**Results:**

A total of 93 MRSA isolates were studied. The vancomycin MICs were 0.75, 1, 1.5 and 2 mg/L for 1 (1.1%), 19 (20.4%), 38 (40.9%), 35 (37.6%) isolates, respectively. During the 3 year period, we observed a significant fluctuation in the rate of MRSA with a vancomycin MIC > 1 mg/L (2007: 86.2%; 2008: 93.3%; 2009: 58.8%, p = 0.002). No MRSA isolate presented a MIC > 2 mg/L.

**Conclusions:**

We were unable to find in our institution data compatible to the presence of vancomycin MIC creep during the study period. This phenomenon seems not to be generalized; as a result each institution should systematically monitor MRSA vancomycin MIC over time.

## Background

According to the Surveillance Network database, which collected laboratory data from 300 clinical microbiology laboratories across the United States, from 1998 to March 2005 with more than 3 million bacterial isolates, *Staphylococcus aureus* was the most prevalent (18.7%) species isolated from inpatient specimens and the second most prevalent (14.7%) species from outpatient specimens [1]. In 2005, MRSA rates were 59.2%, 55%, and 47.9% for strains from non-intensive care unit (ICU) inpatients, ICU, and outpatients, respectively [1].

Although the proportion of *Staphylococcus aureus* bacteraemia due to MRSA is declining in many countries, data from the European Antimicrobial Resistance Surveillance System (EARSS) for 2010 showed that in more than one-third of countries the proportion remained >25% [2]. The highest rates were reported from the Mediterranean countries, with Portugal being the only country showing MRSA rates above 50% [2,3]. The Portuguese data were further confirmed by Melo-Cristino et al. in a recent study [4].

Vancomycin, a glycopeptide antimicrobial, was initially used to treat infections with penicillin-resistant *Staphylococcus aureus* before alternative, less-toxic drugs were introduced [5]. Subsequently this glycopeptide became vital for the treatment of infections with MRSA [5]. Vancomycin is now the 1^st^ line treatment for infections caused by MRSA [6]. However, vancomycin treatment failure in MRSA is not uncommon, even when MRSA is susceptible to vancomycin.

There are a number of types of susceptibility test available. Traditionally, agar disk diffusion has been used to measure glycopeptide susceptibility, but now this method is not regarded as standard, since it does not measure the MIC. This method is not suitable for large antibiotic molecules, such as glycopeptides and daptomycin, because they diffuse too slowly into agar. An alternative method for measuring glycopeptide MIC is broth microdilution – the gold-standard test for measuring antibiotic MICs. Automated susceptibility testing systems are also widely used, but the performance of this methodology for measuring glycopeptide MICs has been frequently questioned. Etest has been developed as an accurate and easier agar plate method. An Etest strip, which contains a gradient of antibiotic, is placed on an inoculated agar plate and the pattern of bacterial growth is examined after 24 hours. Since the Etest uses a gradient of antibiotic concentration, it has greater precision than disc diffusion methods, allowing better ascertainment of the actual MIC.

There is a growing body of evidence showing a sustained increase in the MICs of glycopeptides against *Staphylococcus aureus* strains, a process referred to as “MIC creep”, however with several conflicting results [7-9].

Thus, the primary objective of our study was to evaluate MIC trends for clinical MRSA isolates to vancomycin over a 3-year period in one hospital using the Etest.

## Methods

This study was previously approved by the ethical committee of our institution; no informed consent was needed, according to the ethical principles of the declaration of Helsinki.

### Microorganisms

Clinical MRSA isolates from cultures were collected from sequential individual patients at São Francisco Xavier Hospital, Lisbon, from January 2007 to December 2009. São Francisco Xavier Hospital is a central and university hospital of Lisbon, which belongs to a 900 beds Hospital Centre serving a population of about 935,000 people as a tertiary referral center.

The MRSA isolates were collected mainly from bloodstream, from respiratory tract (tracheal aspirate and bronchoalveolar lavage) and from synovial fluid.

Only one isolate per patient was included in this analysis. For those patients with more than one isolate, only the first isolate was tested. All isolates were identified as *Staphylococcus aureus* according to standard methods.

### Antibiotics evaluated

MICs of vancomycin and oxacillin were determined by the Etest. Oxacillin testing was performed to confirm oxacillin resistance.

### MIC testing methods

For each isolate prior to MIC testing, a single bead was aseptically removed from the Microbank vial and spread onto the surface of trypticase soy agar plates supplemented with 5% sheep blood. These plates were then incubated overnight (18 – 24 h) at 35°C in ambient air. Each isolate was subcultured and incubated overnight for a second time under the same conditions. From these plates, portions of three to five individual colonies were inoculated into 5 mL of physiologic serum and incubated for 18 h. A 0.5 McFarland turbidity standard was used to streak the inoculums onto the surface of a 150 mm Mueller – Hinton II agar plate. The surface of the plate was allowed to dry for 15 min prior to Etest strip application.

The MIC testing was performed using the Etest method, following manufacturer’s guidelines. The antibiotic Etest strips were applied to the agar surface using an Etest applicator and were not moved following application. The MICs were read in 24-48 h. The MIC testing of the organisms was performed over a period of 4 weeks in a single laboratory.

The susceptibility breakpoint was 2 mg/L for vancomycin. The breakpoint for oxacillin resistance was 4 mg/L. Actual Etest MIC values were used for all calculations and analyses and not rounded up to the next highest traditional 2-fold MIC value.

### Statistical analysis

Standard descriptive statistics were used. Continuous variables were reported as mean ± standard deviation.

Continuous variables were analyzed using the parametric unpaired Student’s *t* test, the nonparametric Mann–Whitney *U* test or Kruskal-Wallis H test, according to data distribution. Categorical variables were compared using the Chi-square test.

MIC trends over the 3 years were assessed using non-parametric methods. For the analysis of MIC trends over time, Chi-square test was used.

Tests were performed two-tailed and considered significant when p < 0.05. All statistical tests were performed using SPSS for Windows (version 16.0: SPSS, Chicago, IL, USA) [4].

## Results

During the study period a total of 93 MRSA isolates from 93 patients were collected for analysis. Twenty-nine isolates (31,2%) were recovered in 2007, 30 (32,3%) in 2008 and 34 (36,6%) during 2009.

The majority of MRSA isolates (57%) were collected from bloodstream and from respiratory tract (41,9%) mainly from tracheal aspirate. Only one MRSA was collected from synovial fluid. Clinical and demographic characteristics are presented in Table 1.

**Table 1:** **Clinical and demographic characteristics from the methicilin-resistant *Staphylococcus aureus *(N = 93)**

	***N = 93***
Age, mean (± SD)	76,7 ± 13,0
Gender, F (%)	52 (55,9%)
Department, N (%)	
Emergency department	44 (47,3%)
Medical ward	27 (29,0%)
Intensive care unit	18 (19,3%)
Surgical ward	4 (4,3%)
MRSA, N (%)	
Blood cultures	53 (57%)
Respiratory tract	39 (41,9%)
Others	1 (1,1%)

The lowest MIC was 0.75 mg/L that was observed in only one isolate.

Nineteen isolates (20.4%) had MIC 1 mg/L, 38 (40,9%) isolates had MIC 1.5 mg/L and 35 (37.6%) had MIC 2 mg/L. No MRSA isolate presented a MIC >2 mg/L.

**Figure 1:**
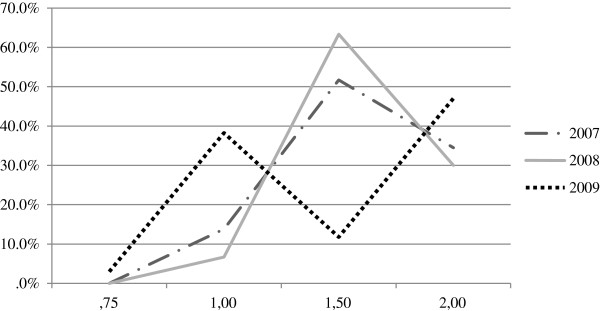
Vancomycin MIC population distribution 2007 – 09.

The MIC distribution for vancomycin is displayed in Figure 1. During the 3 year study period, we observed a significant fluctuation in the rate of MRSA with a vancomycin MIC > 1 mg/L (2007: 86.2%, 2008: 93.3%, 2009: 58.8%). Overall MICs for vancomycin declined during the study period. The MIC trends appeared to either plateau or slightly increase between 2007 and 2008, (p = n.s) but decreased in 2009 (p = 0.002).

## Discussion

Methicillin-resistant *Staphylococcus aureus* is a major pathogen of nosocomial infections and is associated is associated with high mortality rates and healthcare costs [10,11].

Studies reporting vancomycin MIC creep with MRSA have produced conflicting results. Minimum inhibitory concentration creep over time has been noted in some studies but large multicentre surveillance studies have not reported the same type of findings over time [12-14].

The factors involved in the development of reduced susceptibility to vancomycin and subsequent “glycopeptide MIC creep” are not entirely elucidated, but recognition of the phenomenon is important since it may be a precursor to hVISA and VISA [15]. This phenomenon, MIC creep, is of clinical concern because poorer treatment outcomes have been associated with higher vancomycin MICs [9,16-20].

Some authors advocate that combining data from multiple centers can obscure trends that may exist in one institution [14]. Some of these studies utilize less-sensitive traditional susceptibility markers (e.g. percentages, MIC50, MIC90) in their analyses. Vancomycin MIC result also varies by method of testing with Etest results tending to be 0.5–1.5 log_2_ dilutions higher than reference broth method [21-23]. On the other hand, combining data from multiple centers can obscure trends that may exist within a given institution or country as a result of differences in patient populations and drug usage patterns.

In our institution we were unable to find data compatible with the presence of vancomycin MIC creep among MRSA over a 3-year period, on the contrary we found a significant statistical decline in vancomycin MIC creep in 2009.

Our study had some limitations. First, this is a retrospective single center study, possibly introducing a selection bias. Second, the number of patients enrolled was small, making statistical calculations problematic. Third, MICs were only analyzed over a 3-year period, longer periods are needed to a broad statement about MIC trends. However this is the first Portuguese study and in this study we analyzed only one isolate per patient and only isolates in significative samples were included (colonizations were not analyzed).

## Conclusions

In conclusion, though the cause of vancomycin “MIC creep” is unknown, decreased MRSA susceptibility to vancomycin is likely due to overuse as well as to sub-optimal vancomycin dosing. The phenomenon known as MIC creep has been observed only in the last 20 years. Critical evaluation of site of infection, MIC data, aggressive dosing with close monitoring and follow up is warranted with vancomycin therapy. We also advise that all hospitals should monitor their local status of vancomycin MICs in invasive MRSA isolates to screen for the possibility of MIC creep.

## Competing interests

J.S., E.G. and F.M. have no conflicts of interest to declare. P.P. has received honoraria and served as advisor of Gilead, Merck Sharp & Dohme and Pfizer.

## Authors' contributions

JS designed the study, collected the data and wrote the manuscript, EG collected the data and reviewed the manuscript, FM reviewed the manuscript, PP designed the study and reviewed the manuscript. All authors read and approved the final manuscript.
